# Epigenetic modification in Parkinson’s disease

**DOI:** 10.3389/fcell.2023.1123621

**Published:** 2023-06-07

**Authors:** Hao Song, Juanli Chen, Jin Huang, Peng Sun, Yanming Liu, Li Xu, Chuanfei Wei, Xin Mu, Xianjie Lu, Wei Wang, Nan Zhang, Miwei Shang, Mei Mo, Wei Zhang, Hui Zhao, Fabin Han

**Affiliations:** ^1^ The Innovation Institute for Traditional Chinese Medicine, Shandong University of Traditional Chinese Medicine, Jinan, Shandong, China; ^2^ The Institute for Tissue Engineering and Regenerative Medicine, Liaocheng University/The Liaocheng People’s Hospital, Liaocheng, Shandong, China; ^3^ Laboratory of Basic Medical Research, PLA Strategic Support Force Characteristic Medical Centre, Beijing, China; ^4^ Affiliated Yidu Central Hospital, Weifang Medical University, Weifang, China; ^5^ Zhengzhou Revogene Scientific Co., LTD., Zhengzhou, Henan, China

**Keywords:** Parkinson’s disease, epigenetic modificaiton, DNA methylation, histone methylation, histone acetylaion, miRNA

## Abstract

Parkinson’s disease (PD) is a common neurodegenerative disorder caused by genetic, epigenetic, and environmental factors. Recent advance in genomics and epigenetics have revealed epigenetic mechanisms in PD. These epigenetic modifications include DNA methylation, post-translational histone modifications, chromatin remodeling, and RNA-based mechanisms, which regulate cellular functions in almost all cells. Epigenetic alterations are involved in multiple aspects of neuronal development and neurodegeneration in PD. In this review, we discuss current understanding of the epigenetic mechanisms that regulate gene expression and neural degeneration and then highlight emerging epigenetic targets and diagnostic and therapeutic biomarkers for treating or preventing PD.

## Introduction

Parkinson’s disease (PD) is a progressive disorder characterized by resting tremors, rigidity, slow movement, and gait instability. It affects more than 1%–3% of people over 65 years old in the world. Although levodopa replacement and deep brain stimulation can improve PD symptoms, no treatment can stop disease progression (Pringsheim et al., 2021; Zhang et al., 2022; Livingston and Monroe-Duprey, 2023). Molecular studies have characterized that the pathological hallmark of PD is misfolded protein aggregation (Lewy body) mainly composed of α-synuclein (α-SYN), which is coded via the SNCA gene in dopaminergic neurons in substantia nigra (SN) of PD patients (Dodel et al., 2008; Panicker et al., 2021). PD is divided into sporadic cases with unknown causes and familial cases which are attributed to causative mutations in the genes, such as SNCA and Parkin. Overexpression of SNCA is involved in the pathogenesis of familial and sporadic PD.

Since identification of the mutations in the *SNCA* gene in familial PD in 1997, >50 PD-related genes and loci have been identified. These include the PD loci of PARK1-15, such as SNCA, Parkin, Dj-1, and LRRK2, and other PD-associated genes, such as MAPT, GBA, SMPD1, and LRP10 (Nalls et al., 2019; Bandres-Ciga et al., 2020; Day and Mullin, 2021; Song et al., 2022). These studies have shown that the expression products of PD-related genes can interact with cellular molecules in lysosomes and mitochondria to induce apoptosis and neurodegeneration in PD (Coppedè, 2012; Yu et al., 2015; Panicker et al., 2021; Song et al., 2022). In addition to the genetic variations in the PD genes, studies have also demonstrated epigenetic regulations, which can change the expression of mRNAs and proteins associated with the pathogenesis of PD. Epigenetic modifications alter the expression of a gene or chromosomal region without changing the DNA sequence of the gene itself (Pavlou and Outeiro, 2017; Bertogliat et al., 2020; Rittiner et al., 2022).

**FIGURE 1:**
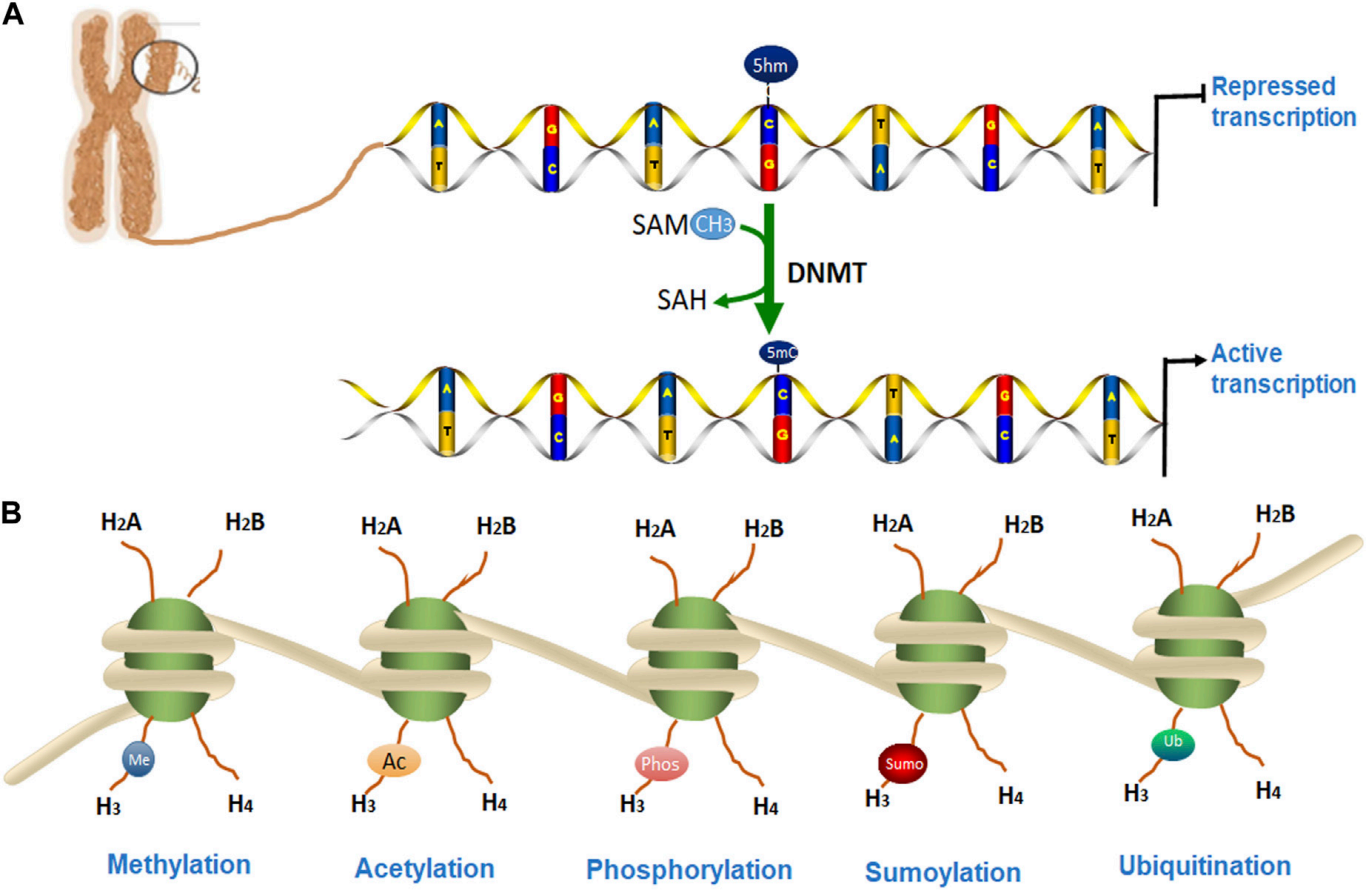
Epigenetic modifications of chromatin. **(A)**. DNA methylation occurs by adding a methyl group to cytosine, thereby converting it to 5-methylcytosine (5-mC) of CpG islands. **(B)**. DNA is packaged into a chromosome as chromatin, which is wrapped around the nucleosome structure and contains two copies each of core histone protein (H2A, H2B, H3, and H4) with protruding N-terminal tails. **(A)** Several post-translational modifications (PTMs) can occur at histone proteins to regulate chromatin structure and gene transcription. Epigenetic PTMs of histones associated with a heterochromatin state include methylation (blue), acetylation (orange), phosphorylation (pink), SUMOylation (red), and ubiquitination (green).

Eukaryotic chromatin is tightly packaged inside the nucleus of the cell. The basic DNA packaging unit in the nucleus is the nucleosome, which is composed of 147 bp DNA wrapped around a histone octamer (two copies of histones H2A, H2B, H3, and H4). Nuclear chromatin regulates various cellular and biological processes by keeping some specific DNA loci in an ‘open’ state where they are accessible to proteins responsible for DNA replication, transcription, and DNA repair, while the other regions of DNA are not accessible. Transcriptionally active chromatin is defined as euchromatin while transcriptionally silent chromatin is referred to as heterochromatin (Grunstein, 1997; Campos and Reinberg, 2009). Some epigenetic alterations have been shown to affect the onset and progression of many degenerative diseases including PD (Jiang et al., 2008; Basavarajappa and Subbanna, 2016). In this review, we discuss the epigenetic mechanisms for PD which includes DNA methylation, the post-translational modification (PTM) of histones, and microRNAs (miRNAs) as shown in Figure 1. We will also summarize some potential epigenetic diagnostic and therapeutic targets for PD.

## DNA methylation in Parkinson’s disease

DNA methylation is the key epigenetic modification by which a methyl group is added to cytosine, converting it to 5-methylcytosine (5-mC) by DNA methyltransferases (DNMTs). This modification occurs on cytosine adjacent to guanines (the CpG islands) and plays a crucial role in regulating gene expression, cellular differentiation, and developmental process. In mammalian cells, DNMT1 is the major regulating enzyme for DNA methylation after replication, while DNMT3a and DNMT3b are essential for the *de novo* methylation predominantly during early development and gametogenesis (Bird, 2002; Andrews et al., 2023). Past studies have demonstrated that the pathogenesis of PD is mainly attributed to the elevated expression of α-synuclein which induces protein aggregates (Lewy bodies) in the middle brain of patients with PD (Espay et al., 2020). Even though the pathogenesis of familial PD is caused by mutations in the PD genes, such as SNCA, LRRK2, and Parkin, for most sporadic patients, the disease is caused because of a combination of mutations in the PD-associated genes and environmental risk factors. Methylation alterations have been found in the promoter/intron 1 of these PD genes, contributing to PD risk (Cai et al., 2011; Panicker et al., 2021; Sohrabi et al., 2023). A recent review demonstrates the genetic and environmental factors leading to PD risk and summarizes epigenome-wide association studies (EWAS) of PD conducted over the past decade. Variants in the *SNCA* gene, exposure to environmental factors, and physical activity are suggested to impact the level of CpG methylation in PD risk genes, thereby contributing to PD pathology (Schaffner and Kobor, 2022).

An earlier genome-wide DNA methylation study detected 2908 CpG islands with differential methylation (DM) in the brain and 3897 CpG islands in the blood of patients with PD (Masliah et al., 2013). Aging-related methylation patterns indicated methylation alterations associated with aging and age-related diseases (Noroozi et al., 2021). Further analysis of the top 1,000 genes with the aging-related methylation pattern identified 10 genes with DM are associated with PD. These genes include SLC12A5, ABCA3, FHIT, FAT1, CPLX2, APBA1, MAGI2, CNTNAP2, ATP8A2, and SMOC2, most of which are related to neural degeneration. This study supported the role of epigenetic alterations as a molecular mechanism for PD (Masliah et al., 2013). A recent study comparing DNA methylation and transcriptome changes in blood samples from patients with PD and matched controls identified 9 genes that were downregulated and 21 genes that were overexpressed in patients with PD. Furthermore, 31 differentially methylated regions (DMRs) were identified, of which 13 regions have the CpG islands that were hypermethylated, whereas 18 regions were hypomethylated in patients with PD. The DMRs were mainly found in the genes of NFYA, DDR1, RING finger ubiquitin ligase (RNF5), acetyltransferase AGPAT1, and vault RNA (VTRNA2-1). This study indicated that more genes are hypomethylated in patients with PD, suggesting methylation alteration could be an accurate and non-invasive molecular biomarker for PD (Henderson et al., 2021). Further analysis for 44 DMRs indicated that hypomethylated regions include the promoter region of NFYA and Discoidin domain receptor (DDR1) which codes for a receptor tyrosine kinase. The DDR1 gene was also hypomethylated in patients with PD. A DDR1 inhibitor was shown to degrade misfolded α-synuclein aggregates and increased dopamine release in animal models with neurodegenerative diseases (Pagan et al., 2019). The hypermethylated CpG sites were identified near the promoter regions in the genes of RNF5, AGPAT1, VTRNA2-1, and CYP1A1. Both RNF5 and AGPAT1 have been associated with neurodegeneration. The hypermethylation was found in 15 CpG sites spanning the genomic region of the vault RNA2-1 (VTRNA2-1) gene which has two-fold increased expression in the frontal cortex of late-stage patients with PD. The gene coding for the key metabolizing enzyme, CYP1A1 was found to be hypermethylated, which may influence Parkin expression and thus is associated with progression of PD. In addition, the M1 polymorphism (T6235C transition) in CYP1A1 is also found to be associated with PD risk (González-Barbosa et al., 2019; Henderson et al., 2021). The dopamine transporter (DAT) gene codes for dopamine transport and functions for the dopamine release in dopaminergic neurons in the SN and striatum of the brain. DNA methylation of six CpG sites in the 5′-UTR of the DAT1 gene was detected in peripheral blood mononuclear cells of 101 sporadic patients with PD compared to 59 healthy controls. As a result, significantly higher methylation at specific CpG2, CpG3, and CpG5 of CpG sites was associated with advanced-stage of patients with PD (Rubino et al., 2020).

Hypomethylation in the promoter region of the *SNCA* gene is usually associated with increased expression. DNA methylation at SNCA intron 1 was shown to affect SNCA transcription initiation. A lentiviral vector is developed by CRISPR-deactivated Cas9 (dCas9) fused with the catalytic domain of DNA-methyltransferase 3A (DNMT3A) for targeted DNA methylation within intron 1 of SNCA. Fusion of the DNMT3A catalytic domain with CRISPR-dCas9 drives DNA methylation. After this vector is delivered to dopaminergic neurons derived from human induced pluripotent stem cells (hiPSC) with the SNCA triplication, the mRNA and protein expression of SNCA were downregulated by increasing DNA methylation at intron 1 of SNCA. DNA methylation at intron 1 decreases the expression of SNCA and reduces cellular protein aggregates of the hiPSC-derived dopaminergic neurons with SNCA triplication, as well as reduced mitochondrial ROS production. This study indicated that the specific DNA target sequence combined with the CRISPR-dCas9 technology can be a novel epigenetic-based therapeutic approach for PD (Kantor et al., 2018).

To identify methylation of α-synuclein (SNCA) and leucine-rich repeat kinase 2 (LRRK2) in leukocytes in PD, bisulfite specific PCR-based sequencing was used to detect methylation of CpG islands in the promoter (CpG-1) and intron 1 (CpG-2) of *SNCA* and *LRRK2*. Although there were no methylation differences in CpG-1 of *SNCA* or the *LRRK2* promoter, the second, fourth, and ninth CpG sites of the 14 CpG sites of CpG-2 were significantly hypomethylated in patients with PD. Notably, decreased methylation in *SNCA* is associated with early-onset PD and increased SNCA mRNA in PD (Tan et al., 2014). The Rep1 polymorphism of *SNCA* is associated with sporadic PD. A study analyzing the relationship between the methylation status of *SNCA* intron-1 and Rep1 polymorphism in peripheral blood monocyte cells (PBMCs) from 100 sporadic patients with PD and 95 normal controls found that hypomethylation in *SNCA* intron-1 was associated with increased SNCA expression. Hypomethylation in the SNCA intron-1 was also identified in *postmortem* brains from sporadic patients with PD and DNA methylation was associated with the Rep1 polymorphism (Ai et al., 2014). Another study analyzed the methylation status of 72 blood and 24 cerebral cortex samples from patients with PD by detecting CpG islands in intron 1 of *SNCA* using methylation-sensitive restriction enzyme digestion and quantitative polymerase chain reaction (PCR). Patients with PD showed significant hypomethylation compared with controls (Pihlstrom et al., 2015). An earlier next-generation study using 454 GS-FLX sequencing to analyze methylation in *SNCA* in 15 patients with Lewy body disease (LBD) and 187 controls indicated that the overall methylation levels in the promoter and intron 1 regions of SNCA were higher in LBD patients (de Boni et al., 2011). A study investigating if altered DNA methylation in SNCA intron 1 is a mechanism that results in PD and dementia with Lewy bodies (DLB) found that Dnmt1 expression is reduced in the nuclear compartment of *postmortem* brain samples from PD and DLB patients as well as in brain samples from α-synuclein transgenic mouse models. After Dnmt1 is sequestered from the nucleus to cytoplasm, global DNA hypomethylation occurs in human and mouse brains, involving CpG islands in intron 1 of the *SNCA*, *SEPW1*, and *PRKAR2A* genes. Furthermore, reduced levels of nuclear Dnmt1 were partially rescued via overexpression of Dnmt1 in cultured neuronal cells and brains of α-synuclein transgenic mice, suggesting an epigenetic dysregulation mechanism in PD and DLB (Desplats et al., 2011).

Over the past decade, studies have shown that 5-hydroxymethylcytosine (5 hmC) is an epigenetic modifier of various neurodegenerative diseases, including PD. 5 hmC is the oxidation product of 5-methylcytosine (5 mC) ten-eleven translocation (TET) enzymes and generally upregulates gene expression, which is different from the effects of 5 mC (Al-Mahdawi et al., 2014). The 5 hmC level is ∼10% that of 5 mC in the mammalian genome. High levels of 5 hmC are found in the central nervous systems, which is ∼40% as abundant as 5 mC (Sherwani and Khan, 2015). A study that performed sequence analysis for 12 PD genes (including *SNCA*, PARKIN, and *TET*) related to DNA methylation and hydroxymethylation in 1,657 patients and 1,394 controls identified *TET1* as a major gene in PD, further suggesting that hydroxymethylation might play an important role in PD (Shu et al., 2019). To analyze alterations in DNA methylation (5mcC) and hydroxymethylation (5 hmC) in PD, a recent genome-wide study screened 5 mC and 5 hmC in the substantia nigra of brains from patients with PD and identified 4,119 differentially hydroxymethylated regions (DhMRs) compared to controls. Further analysis revealed that the PD-associated DhMRs are associated with several signaling pathways, including phospholipase D (PLD), cAMP, and Rap1, that regulate neurogenesis and neuronal differentiation. Notably, one of the 5 hmC-modulated genes, PLD1, was found to modulate toxicity of α-synuclein in a *Drosophila* model of PD. This study demonstrated that 5 hmC may act as an independent epigenetic mechanism contributing to the pathogenesis of PD (Min et al., 2022). A genome-wide study identified elevated hydroxymethylation levels in cytosine modifications at enhancers in patients with PD compared to controls. Patients with PD also showed epigenetic transcriptional upregulation of TET2, which is the main enzyme modifying cytosine in DNA sequences. Moreover, inactivation of Tet2 in mice has been shown to prevent MPTP-induced dopaminergic neuronal loss in the substantial nigra of mouse brains. This study demonstrated that widespread epigenetic dysregulation of enhancers of neurons in PD mice may be mediated via increased TET2 expression, suggesting that downregulation of Tet2 could be neuroprotective and an epigenetic therapeutic target for PD (Marshall et al., 2020). A summary of studies investigating DNA methylation for PD is listed in Table 1.

**TABLE 1:** DNA methylation.

DNA methylation site	Effect	Gene/Target	Study model	Tissue/Study method	Main results	References
Hypomethylation,5 hmC	↓	SNCA(CpG2/intron1)	PD (n = 91), Ctrl (n = 52)	Blood, Brain/methylation array technology	Error Collapse α-synuclein	Yu et al. (2015), Eryilmaz et al. (2017), Song et al. (2022)
Hypomethylation,5 hmC	↓	Parkin	PD (n = 91), Ctrl (n = 52)	Blood, Brain/specific-PCR (MSP)	Mitochondrial dysfunction leads to apoptosis of dopaminergic neurons	Blanch et al. (2016), Eryilmaz et al. (2017)
		(CpG17/Intron 2)				
Hypomethylation,5 hmC	↓	DDR1	PD (n = 15), Ctrl (n = 15)	Blood/methylation array technology	Tyrosine kinase	3 (Henderson et al., 2021)
		(CpG8/Intron1)				
Hypermethylation,5 hmC	↑	VTRNA2-1(CpG15/Intron1)	PD (n = 15), Ctrl (n = 15)	Blood/methylation array technology	Neural tube defects	González-Barbosa et al. (2019)
Hypermethylation,5 hmC	↑	CYP1A1	PD (n = 15), Ctrl (n = 15)	Blood/methylation array technology	Neurotoxin enrichment	Henderson et al. (2021)
		(CpG10/Intron1)				
Hypermethylation,5 hmC	↑	DAT	PD (n = 101), Ctrl (n = 59)	Blood/methylation array technology	Dopamine transporter	Rubino et al. (2020)
		(CpG5/Intron1)				
Hypomethylation,5 hmC	↓	MAPT	PD (n = 101), Ctrl (n = 59)	Blood/methylation array technology	Tau protein increase in cerebrospinal fluid	Han et al. (2017), Song et al. (2022)
		(CpG2				
		/promoter)				
Hypomethylation,5 hmC	↓	NPAS2	PD (n = 101), Ctrl (n = 59)	Blood/methylation array technology	Clock gene neuron	Blanch et al. (2016)
		(CpG1,2/Promoter)				
Hypomethylation,5 hmC	↓	PGC-1	Mice	Blood/methylation array technology	Mitochondrial dysfunction	Blanch et al. (2016)
		(CpG13,14/Intron1)				

### Histone methylation in Parkinson’s disease

The N-terminal amino acid residues of histones can be modified to activate or suppress expression of specific genes (Smolle and Workman, 2013). Histone methylation is preferentially at arginine or lysine residues of H3 and H4 histones to regulate the response to DNA damage. The arginine or lysine residues can be subjected to monomethylation or dimethylation by histone methyltransferase (KMTs) or histone demethylase (KDMs). Studies have shown that methylation of histone H3 on lysine 4 (H3K4), lysine 14 (H3K14), lysine 36 (H3K36), lysine 79 (H3K79), or arginine 17 (H3R17) are mainly involved in transcriptional activation of the related genes whereas methylation of histone H3 at lysine 9 (H3K9), lysine 27 (H3K27) or histone H4 on lysine 20 (H4K20) is often related to transcriptional repression of the related genes (Pavlou and Outeiro, 2017; Di Nisio et al., 2021). The common H3K4 methylation is mainly associated with active gene expression and this methylation is catalyzed by several proteins such as SET1A, SET1B, MLL1, MLL2, MLL3, MLL4, and ASH1(Bernstein et al., 2002).

The histone methylation of the SNCA gene regulates the expression of α-synuclein (α-SYN) in PD. Overexpression of α-SYN in flies and SH-SY5Y neuroblastoma cells can enhance histone methylation of H3K9 me1, H3K9 me2, and H3K9 me2-target genes such as L1cam and Snap25. After cells are treated with euchromatic histone-lysine N-methyltransferase 2 (EHMT2) inhibitor UNC0638, the mRNA expressions of L1CAM and SNAP25 were found to be restored, indicating that α-SYN enhances H3K9 methylations via ΕΗΜΤ2 to result in elevated H3K9 me2 and eventually impairs the synaptic activity of neurons in PD (Sugeno et al., 2016). To study the neuroprotective effect of GSK-J4, a potent histone demethylase inhibitor of H3K27 me3/me2 and H3K4 me3/me2, dopamine neurons were pre-treated with GSK-J4 and then treated with HO_2_O_2_ and 6-OHDA. GSK-J4 was found to be selectively reduced by HO_2_O_2_ and 6-OHDA-induced neural cell death *in vitro*. Furthermore, GSK-J4 also protected dopaminergic neuron loss and motor defects in 6-OHDA-induced PD rats. This study indicated that GSK-J4 rescued the abnormal changes of histone methylation, H3K4 me3 and H3K27 me3, during 6-OHDA treatment, suggesting that modification of histone methylation by small molecules may be a potential therapeutic approach for the protection and treatment of PD and other neurodegenerative diseases (Mu et al., 2020; Wang et al., 2021).

A recent study analyzed the histone post-translational modifications (PTMs) of genes in SN tissues from two patients with PD and suggested two important histone methylations of H3K4 me3 and H3K27 me3 are associated with the promoter region of SNCA, which is in a range of approximately 1 kb–1.5 kb downstream of the transcription start site. The H3K4 me3 is to promote transcription initiation and was found to be increased in the brains of patients with PD. However, H3K27 me3 is associated with the repression of the SNCA gene. These findings indicated that abnormal histone methylation affects the gene expression of SNCA and the pathogenesis of PD (Guhathakurta et al., 2021). A summary of the studies investigating histone methylation for PD is listed in Table 2.

**TABLE 2:** Histone modifications.

Histone modification	Effect	Gene/Target pathway	Study model	Tissue/Study method	Main results	References
Methylation, (H3K9me1, H3K9me2	↑	SNCA,L1cam, Snap25	*Drosophila*, SH-SY5Y cell	Overexpression of α-SYN	impaired synaptic activity	Sugeno et al. (2016)
Methylation, (H3K4me3 and H3K27me3)	↓	histone demethylase	dopaminergic neurons; 6-OHDA-induced PD rats	GSK-J4, a histone demethylase inhibitor	neuroprotection *via* epigenetic mechanism	Mu et al. (2020)
Methylation (H3K4me3)	↑	SNCA	PD patients	SN		Guhathakurta et al. (2021)
Methylation (H3K27me3)	↓	SNCA	PD patients	SN	Significantly elevated methylation	Guhathakurta et al. (2021)
Acetylation, AcH3-Lys9	↓	AGK2,SIRT2	N27 dopaminergic neurons; PD patients	SN,Immunoblotting	Induce Neural degeneration	Harrison et al. (2018)
Acetylation (H3K27ac)	↑	SNCA	PD patients	SNImmunoblotting	increased expression of α-synuclein	Guhathakurta et al. (2021)
Acetylation,H2BK15ac, H3K9/14 ac, H3K27ac, H3K56ac and H4K12ac	↑	SNCA, PARK7, PRKN and MAPT	PD patients	fresh-frozen brain tissue; Immunoblotting and RNA sequencing	Neurodegeneration	Toker et al. (2021)
Phosphorylation on HDAC3	↑	HDAC3 at Ser-424; deacetylation of Lys-5 and Lys-12 on histone H4	SH-SY5Y cells and mouse embryonic fibroblasts (MEFs)	Immunoprecipitation and western blots	Increase 6-OHDA-induced cell death	Han et al. (2017)
Ubiquitination H2AK119ub	↑	H2AK119ub/SCNA	neural stem cells	*In vitro* cell culture	Increase degradation of protein aggregates	Srivastava et al. (2021)

## Histone acetylation in Parkinson’s disease

Histone acetylation is the addition of an acetyl group at lysine residues by histone acetyltransferases (HATs), which is associated with transcriptional activation whereas histone deacetylation usually represses transcription by removing an acetyl group from the acetylated histones, which is catalyzed by histone deacetylases (HDACs) (Barski et al., 2007). The histone deacetylase 3 (HDAC3) is a transcriptional repressor to suppress p53-dependent apoptosis. PINK1 is an autosomal recessive gene for PD and the PINK1 protein with phosphorylase activity can bind to the promoter region of HDAC3 and increase its deacetylase activity through phosphorylation of HDAC3 in neuronal cell lines. PINK1 is also found to increase the binding of phosphorylated HDAC3 to p53 to inhibit p53 acetylation and, thus alleviate p53-mediated neuronal apoptosis. Mutations in PINK1 result in mutant PINK1 protein that cannot phosphorylate HDAC3, which leads to a dysfunction of HDAC3 and increased p53-dependent neuronal apoptosis and neurodegeneration in PD (Choi et al., 2015; Labbé et al., 2016). PD is not only associated with the loss of dopaminergic neurons but also has activated microglia in the SN of the brain. Examining histone acetylation in SN of post-mortem patients with PD and age-matched controls showed a significant increase in acetylated histone residue of AcH3-K9, AcH2A-K5, AcH2B-K15, and AcH4-K5 in patients with PD compared with that of control individuals. However, the histone acetylation in AcH2A-K5, AcH3-K9, and AcH4-K5 was not found to increase in the cerebellar cortex from the patients with PD, indicating that histone acetylation is specifically affecting the SN region (Park et al., 2016). Some studies have demonstrated that alterations in lysine acetylation and deacetylation of histone and nonhistone proteins, which are catalyzed by related lysine acetyltransferases (KATs) and lysine deacetylases (KDACs, respectively, are present in patients with PD and various PD models. An increased level of histone acetylation could be caused because of downregulation of KDACs, including HDAC1, HDAC2, HDAC4, HDAC6, and SIRT1.

A comprehensive analysis of histone acetylation in the SN in the brains of normal individuals, early and late-stage patients with PD, demonstrated that histone acetylation is increased with disease progression in patients with PD, which is likely caused by both degenerating dopaminergic neurons and activated microglia (Harrison et al., 2018). A study has identified transcriptional adapter 2-alpha (TADA2a) as a novel binding partner of α-syn in Lewy bodies. As a p300/CBP-associated factor, TADA2a is associated with the acetylation of histone H3/H4. The A53T mutation in SNCA significantly decreased acetylation of histone H3 in cultured SH-SY5Y cells and histone H3 acetylation is also found to be reduced in the striatum and substantia nigra of PD mice injected with α-synuclein -folded fibrils (PFFs). PFF decreased the expression of TADA2a in the striatum and SN of α-syn PFF-induced PD mice. This study indicated that histone modification may be associated with the α-syn-mediated pathogenesis of PD (Lee et al., 2021). Another study examined the histone modifications in post-mortem brains of patients with PD and controls. As a result, H3K4 me3 was significantly increased at the promoter of SNCA in the SN of patients with PD. To investigate how H3K4 me3 regulates α-synuclein expression, a CRISPR/dCas9-based locus-specific H3K4 me3 demethylating vector was established to reduce H3K4 me3 at SNCA promoter and was shown to decrease mRNA and protein expression of α-synuclein, both in the SH-SY5Y cells of neuronal cell line and dopaminergic neurons derived induced pluripotent stem cells (iPSC) of patients with PD (Guhathakurta et al., 2021). Comprehensive analysis with chromatin immunoprecipitation sequencing (ChIP-seq), immunoblotting, and RNA sequencing identified histone sites with altered acetylation in fresh-frozen brain tissues of 28 patients with PD and 21 control individuals (Toker et al., 2021). This analysis demonstrated a significant acetylation increase in H2BK15ac, H3K9/14 ac, H3K27ac, H3K56ac, and H4K12ac in PD. In addition, H3K27 hyperacetylation was also observed in the PD striatum and cerebellum. The histone acetylation in SIRT1 and SIRT3 genes is also upregulated, but it is not for SIRT2, indicating that sirtuins can modulate the acetylation of this residue. Importantly, this study identified H3K27-hyperacetylated regions in more than 20 genes linked to familial or sporadic forms of PD. H3K27 hyperacetylation is involved in PD-related genes including SNCA, PARK7, PRKN, and MAPT (Dickson, 2018; Lim et al., 2019). The hyperacetylated region was found to be in the enhancer region of the SNCA gene, which was previously shown to be affected by both genetic sequence variation (Soldner et al., 2016) and drug exposure in PD (Toker et al., 2021). A summary of studies investigating histone acetylation for PD is listed in Table 2.

### Other chromatin remodeling in Parkinson’s disease

In addition to histone methylation and acetylation as discussed above, other modifications on histones also regulate cell mitosis, DNA repair, and transcription initiation. The histone phosphorylation, ubiquitination, and other modifications in PD genes are also regulating neurodegeneration and cell death in PD. The SNCA promoter interacts with histones in the nucleus, which was shown to accelerate fibrillation and toxicity of α-synuclein. In addition, α-synuclein is also interacted with SIRT (a deacetylase) to bind to histones and suppress the acetylation of histone H3. Furthermore, the mutated p.A30P and p.A53T of α-synuclein were found to increase nuclear localization of misfolded α-synuclein and formation of fibrillation leading to PD (Kontopoulos et al., 2006).

Phosphorylation of HDAC is shown to increase the pathogenesis of PD. LRRK2 is one of the major causative genes for autosomal-dominant familial PD and possesses guanosine triphosphatase (GTPase) and kinase activities. LRRK2 was demonstrated to directly phosphorylate HDAC3 at Ser-424 to increase the activity of HDAC3 and promoted nuclear translocation of HDAC3. The phosphorylated HDAC3 in the nuclei of neural cells was found to be increased in SN of 6-OHDA-induced PD rats. Thus, the gain of function mutations in LRRK2 promoted neural cell death in 6-OHDA-induced PD rats via modification of HDAC3 in the nucleus of the dopaminergic neurons (Han et al., 2017).

Histone ubiquitination is associated with the degradation of α-synuclein-based protein aggregation in PD. Epigenetic polycomb repressor complex-1 subunit (BMI-1) regulates the self-renewal and differentiation process of neural stem cells. Reduced expression of BMI-1 affects proliferation and mitochondrial homeostasis of neural stem cells and thus promotes neurodegeneration and suppresses neuro in PD. Once BMI-1 is phosphorylated, it induces the accumulation of phosphorylated α-synuclein at serine 129 (pα-SYN). The accumulated pα-SYN is complexed with BMI-1 for ubiquitin-dependent proteasomal degradation to alleviate protein aggregation. This study indicated that increasing ubiquitination of histone 2A at lysine 119 (H2AK119ub) can promote the degradation of α-synuclein-formed aggregates and have a potential therapeutic effect in PD (Srivastava et al., 2021).

SUMOylation is another epigenetic control mechanism of transcription in which small ubiquitin-related modifier (SUMO) proteins are added to lysine chains of histones or other proteins. In humans, the SUMO protein family has four isoforms termed SUMO-1 (also known as UBL1, GMP1, SENTRIN, and SMT3C), SUMO-2 (SMT3A), SUMO-3 (SMT3B), and SUMO-4 (Filosa et al., 2013). Histone SUMOylation usually accounts for a small percentage of histone proteins and plays a repressive role in gene expression of transcription (Ramazi et al., 2020). The SUMOylation process is reversible and regulates many development-related processes including signal transduction, subcellular localization, and protein aggregation (Gareau and Lima, 2010). Dysregulation of SUMOylation is associated with various developmental defects and conditions, such as neurodegenerative diseases and cancer (Kim et al., 2011; Mandel and Agarwal, 2022). SUMOylation of α-synuclein was shown to enhance the formation of α-synuclein aggregates to promote the neurodegeneration of PD. A study indicated that the SUMO ligase, PIAS2 can stimulate SUMOylation of α-synuclein and overexpression of PIAS2 increased SUMOylation and cellular translocation of α-synuclein to form the synuclein aggregates in dopaminergic neurons (Rott et al., 2017). A recent study showed that the dopamine transporter (DAT) is associated with SUMO1 in rat dopaminergic cells (N27) and human embryonic kidney cells (HEK)-293 cells overexpressing DAT. Overexpression of SUMO1 and the Ubc9 SUMO-conjugate induced SUMOylation of DAT and decreased DAT ubiquitination and degradation. Ubc9-mediated SUMOylation also promoted expression of DAT in the cellular membrane and the dopamine uptake capacity of dopaminergic cells. Overall, this study indicated that SUMOylation is a novel mechanism to regulate DAT expression and dopamine uptake in dopamine neurons, suggesting that the SUMO pathway—including SUMO1, SUMO2, Ubc9, and DAT SUMOylation—is a potential therapeutic target for PD (Cartier et al., 2019)**.** A summary of studies investigating other histone modifications for PD is provided in Table 2.

### RNA-based mechanisms of gene regulation

In addition to regulating DNA transcription, methylation controls mRNA expression (Wang et al., 2014). N6-methyladenosine (m6A) and 5-methylcytosine (m5C) are two common post-transcriptional methylation modifications of mRNA. Adenosine methylation is usually observed in brain tissues. m6A methylation can interfere with signaling pathways in neuronal development by downregulating the expression of neuronal mRNAs (Satterlee et al., 2014). In MPTP-induced PD mice model, the mRNA and protein of m6A regulators in the SN and striatum are reported to be differently expressed: m6A regulatory proteins, including ALKBH5 and IGF2BP2, were upregulated in the SN, whereas YTHDF1 and FMR1 were downregulated. In the striatum, FMR1 and CBLL1 were upregulated, whereas IGF2BP3, METTL3, and RBM15 were downregulated. mRNA expression of these genes was partially in accordance with protein changes. These findings indicate that m6A regulators may participate in PD pathogenesis (Yu et al., 2022). Expression of 5-mC and 5-hmC was analyzed from paraffin-embedded brain tissue samples of patients with PD and controls using immunohistochemistry methods. The results showed significant upregulation of 5-mC, but not 5-hmC, in cortical brain sections of patients with PD. However, there were no significant differences in 5-mC or 5-hmC in the brain stem and SN between patients with PD and controls. Of note, 5-hmC was significantly upregulated in the cerebellar white matter of patients with PD, although 5-mC did not change in this brain area. Taken together, these findings indicate that 5-mC and 5-hmC likely play important roles in the pathogenesis of PD (Kaut et al., 2019).

MicroRNAs (miRNAs, or miRs) are small non-coding RNAs with 20–22 nucleotides and are involved in regulating mechanisms of neurodegenerative diseases including PD. The specificity of the miRNAs for targeting specific genes is determined by the miRNA sequence at the 5′-end (bases 2–8) of the miRNAs. As epigenetic regulating factors, miRNAs mainly control the mRNA translation by binding to 3′untranslated regions of mRNAs (Zhang et al., 2019). A lot of miRNAs have been shown to epigenetically regulate the gene expression of PD-associated genes and control the disease progression (Selvakumar et al., 2022). A review study reported that more than 80 miRNAs were reported to be altered in different tissues of patients with PD, of which 15 miRNAs were upregulated or downregulated in the 3 tissue samples of brain, cerebrospinal fluid (CSF), and blood of patients with PD as well as in animal models with PD by RNA sequencing, microarray, and microRNA qPCR analysis from 34 reported screening studies. These 15 miRNAs include miR-1, miR-16, miR-24, miR-26, miR-28, miR-29, miR-30, miR-126, miR-151, miR-200, miR-301, miR-331, miR-374, miR-485 and Let-7 (Goh et al., 2019). Some of the important miRNAs related to PD are discussed here.

A study compared 733 miRNAs in the SNs of 8 patients with PD to that of 4 control individuals and showed that 10 miRNAs are downregulated in PD through TaqMan low-density array analysis. Further analysis indicated that miR-135b was the most downregulated one in SN tissues of patients with PD (Cardo et al., 2014).

miRNAs can regulate the expression of PD genes such as SNCA, LRRK2, and Parkin to be involved in the pathogenesis of PD. miRNA-7 was shown to repress expression of α-Syn by binding to the 3′-untranslated region (UTR) of the mRNA to increase autophagy in differentiated ReNcell VM cells of human neural progenitor cells to degrade the fibrils of α-Syn aggregates in the neural cells. This mechanism for miRNA-7 to reduce α-Syn levels provides a therapeutic target for PD and other alpha-synucleinopathies (Choi et al., 2018). LRRK2 was shown to be upregulated in the brains of patients with PD, but there is no significant difference in the expression level of LRRK2 mRNA between the control and PD cases, indicating the overexpression of LRRK2 may be induced by post-transcriptional modification of LRRK2 mRNA in PD brains. miR-205 was reported to suppress expression of LRRK2 through binding to 3′- UTR of the LRRK2 gene. In addition, overexpression of miR-205 in cultured primary neural cells improved neurite outgrowth of neurons expressing a mutant LRRK2, R1441G, indicating that miR-205 may be used as the therapeutic target to suppress the abnormal upregulation of LRRK2 protein in PD (Cho et al., 2013).

The chronic inflammation induced by microglial activation is a mechanism leading to the neurodegeneration of PD. MicroRNA-124 usually promotes the neurogenesis of the human brain and was found to be downregulated in the 1-methyl-4-phenyl-1,2,3,6-tetrahydropyridine (MPTP)-induced mouse model of PD (Angelopoulou et al., 2019). miR-124 was also found to regulate the microglial inflammatory response by targeting p62 and p38 in PD. In cultured microglial cells, overexpression of miR-124 could suppress the expression of p62 and p-p38 and could decrease the activation of microglial cells in the SN of the MPTP-induced PD mouse model. This study suggests that miR-124 could be a potential therapeutic target for PD (Yao et al., 2019).

In addition to promoting the degradation of α-syn, two miRNAs were found to affect the toxicity of a-synuclein aggregation by regulating phosphorylation and acetylation to affect the neurotoxicity of α-synuclein. miRNA-26a directly downregulated the expression of death-associated protein kinase 1 (DAPK1), and DAPK1 could promote the phosphorylation of a-synuclein. Suppressing miR-26a or upregulating DAPK1 results in α-synuclein aggregation, cell death of dopaminergic neurons, and motor disabilities in wild-type mice (Su et al., 2019). In addition, SIRT2 was identified to catalyze the deacetylation of α-synuclein. miRNA-486-3p can bind to the 3′UTR of SIRT2 and influence the translation of SIRT2. Thus, miRNA-486-3p (miR-486-3p) may affect α-synuclein -induced aggregation and toxicity by decreasing the expression of SIRT2 (Wang et al., 2018).

Nod-like receptor protein 3 (Nlrp3) functioned in regulating inflammatory cytokines.

miRNA-190 was shown to target the 3′ UTR of Nlrp3 mRNA and inhibited neuroinflammation in the MPTP-induced mouse model of PD and BV2 of microglial cells. When the miRNA-190 was overexpressed, the expressions of pro-inflammatory markers, such as iNOS, IL-6, TNF-α, and TGF-β1, were inhibited and the anti-inflammatory mediator such as IL-10 was increased. Furthermore, upregulation of miRNA-190 inhibited activation of microglial cells and thus prevented inflammation and attenuated the tyrosine hydroxylase-neuron loss in SNpc in MPTP-induced PD mice. Thus, miRNA-190 inhibits inflammation reaction and activation of Nlrp3 to play protective roles in the MPTP-induced PD mouse model (Sun et al., 2019).

Nuclear receptor related 1 (Nurr1) is one of the key transcription factors to regulate the development and maturation of dopaminergic neurons by associating with Pitx3, LMXA1, and FOXA2 (Grimes et al., 2006). miRNA-132 was shown to downregulate Nurr1. After the miRNA-132 and Nurr1 in blood samples of patients with PD and healthy controls were examined by RT-qPCR, the plasma miRNA-132 of PD was significantly higher than those in controls. But Nurr1 was significantly decreased in the blood of patients with PD compared with controls. Further analysis demonstrated that a negative correlation between the decreased Nurr1 protein level and the elevated miRNA-132 level in PD indicated that the combination of miR-132 and the Nurr1 might be a potential biomarker for the diagnosis of PD (Yang et al., 2019).

## Conclusion and future challenges

Epigenetic modifications may be involved in the pathogenesis of PD through DNA methylation, acetylation, phosphorylation, and ubiquitylation of histones in chromatins and miRNAs. Epigenetic changes make distinct contributions to neurodegeneration and neural death in PD by regulating gene expression. Although exact epigenetic mechanisms of PD progression are not completely known, current findings indicate critical effects of epigenetic modifications in PD. Some epigenetic alterations can be detected in specific tissues and blood samples from patients with PD and PD animal models, suggesting that epigenetic modulators may be useful biomarkers for PD diagnosis and therapy. Although many studies have revealed different epigenetic mechanisms in PD, epigenetic alterations have not yet been translated into biomarkers suitable for clinical use. One reason may be the difficulty in obtaining *postmortem* brain tissues from patients with PD. Another reason is that DNA methylation may show different patterns in different brain regions of patients with PD. Thus, more studies are needed to determine specific methylation patterns of blood samples from patients with PD as biomarkers. Future challenges are to identify new epigenetic targets to elucidate the precise epigenetic mechanisms in the different pathological stages of PD and to translate epigenetic modulators into novel biomarkers for PD diagnosis and therapy.
